# Socio-demographic disparities in liver cancer mortality in China: a national analysis from 2015 to 2021

**DOI:** 10.3389/fonc.2026.1757881

**Published:** 2026-02-03

**Authors:** Heya Jing, Xiaofeng Xu

**Affiliations:** 1 Liver Transplant Center, Transplant Center, West China Hospital, Sichuan University, Chengdu, China; 2 West China School of Nursing, Sichuan University, Chengdu, China

**Keywords:** age-standardized mortality rate, China, health disparities, liver cancer, mortality, population aging

## Abstract

**Introduction:**

Liver cancer remains a leading cause of cancer-related mortality in China. Analyses based solely on crude mortality rates may obscure temporal trends and inequalities in the context of rapid population aging. This study examined recent trends and socio-demographic disparities in liver cancer mortality in China using age-standardized analyses.

**Methods:**

We conducted a population-based analysis using data from the China National Mortality Surveillance System covering all 31 provincial-level regions of mainland China from 2015 to 2021. Liver cancer deaths were identified using ICD-10 code C22. Mortality rates were calculated for adults aged ≥20 years. Age-standardized mortality rates (ASMRs) were estimated by direct standardization to the 2010 China standard population. Temporal trends were assessed using joinpoint regression to estimate annual percent change (APC) and average annual percent change (AAPC). Disparities by sex, urban–rural residence, and geographic region were quantified using rate differences (RD), rate ratios (RR), and an index of disparity. Decomposition analysis was applied to assess the contributions of population aging and changes in age-specific mortality rates to crude mortality trends.

**Results:**

Between 2015 and 2021, a total of approximately 470,000 liver cancer deaths were recorded. The crude mortality rate changed modestly, whereas the ASMR declined steadily from 30.16 per 100,000 in 2015 to 23.60 per 100,000 in 2021. Joinpoint regression showed a significant overall decline in ASMRs (AAPC −4.0%, 95% CI −5.2% to −2.8%), with no statistically significant joinpoints detected. Throughout the study period, males had substantially higher ASMRs than females (2021: 36.9 vs. 13.8 per 100,000; RR = 2.67). Rural areas consistently exhibited higher ASMRs than urban areas, with the rural–urban RD widening from 6.4 per 100,000 in 2015 to 8.5 per 100,000 in 2021. Regionally, ASMRs declined more rapidly in Eastern China (29.7 to 22.6 per 100,000) than in Central (31.1 to 29.8 per 100,000) and Western China (30.4 to 28.7 per 100,000), resulting in increasing regional disparity. Age-specific mortality increased sharply with advancing age. Decomposition analysis indicated that population aging increased crude mortality, partially offset by reductions in age-specific mortality rates.

**Conclusions:**

Liver cancer mortality in China declined from 2015 to 2021 after age standardization; however, substantial and widening disparities by sex, urban–rural residence, age, and region persist. These findings highlight the importance of age-standardized and equity-focused approaches to liver cancer prevention and control.

## Introduction

Primary liver cancer is one of the leading causes of cancer-related death worldwide and remains a major public health challenge in China. According to the Global Burden of Disease and GLOBOCAN estimates, China accounts for a substantial proportion of global liver cancer mortality, reflecting both historical and ongoing exposure to major etiologic risk factors (1, 2). Despite notable progress in prevention and clinical management, liver cancer continues to impose a heavy mortality burden, particularly among older adults and socially disadvantaged populations.

Chronic infection with hepatitis B virus (HBV) has long been recognized as the dominant cause of liver cancer in China, owing to its high historical prevalence (3). Over the past three decades, large-scale public health interventions—including universal infant HBV vaccination, catch-up vaccination programs, and expanded access to antiviral therapy—have substantially reduced HBV prevalence in younger cohorts (4–6). Nevertheless, the mortality burden of liver cancer remains high, in part because of population aging and the long latency between HBV infection and hepatocarcinogenesis (7). In addition, other etiologic factors, such as hepatitis C virus (HCV) infection, alcohol-related liver disease, and metabolic-associated steatotic liver disease, are contributing increasingly to liver cancer mortality, particularly in urban and economically developed regions (8–10).

Marked sociodemographic disparities in liver cancer outcomes have been reported both globally and within China. Previous studies have consistently shown higher incidence and mortality rates among men compared with women, reflecting differences in biological susceptibility, behavioral risk factors, and health-seeking behaviors (11–13). Urban–rural disparities are also evident, with rural populations experiencing higher mortality and poorer survival, likely due to delayed diagnosis, limited access to specialist care, and lower uptake of surveillance and antiviral treatment (14, 15). Furthermore, substantial geographic heterogeneity exists across regions of China, which may reflect variations in socioeconomic development, healthcare infrastructure, environmental exposures, and historical patterns of HBV endemicity (16, 17).

Monitoring temporal trends and disparities in liver cancer mortality is essential for evaluating the effectiveness of prevention and control strategies and for guiding equitable resource allocation. National mortality surveillance systems provide a valuable platform for such analyses, allowing assessment of long-term trends across population subgroups. However, many previous reports have focused on crude mortality rates or broad national averages, which may obscure important changes in age structure and mask widening or narrowing inequalities across sociodemographic groups (18). Age-standardized analyses and formal disparity metrics are therefore critical to ensure valid comparisons over time and between regions.

The coronavirus disease 2019 (COVID-19) pandemic introduced additional uncertainty into cancer control efforts. Disruptions to routine healthcare services, delays in diagnosis and treatment, and competing mortality risks may have affected cancer outcomes during this period (19, 20). For liver cancer, which often progresses rapidly and relies on timely surveillance and intervention, even short-term disruptions could have measurable effects on mortality trends. Evaluating recent data that span the pandemic period is thus particularly relevant.

In this context, we used nationally representative data from the China National Mortality Surveillance System to examine liver cancer mortality in China from 2015 to 2021. Our objectives were to: (1) characterize temporal trends in liver cancer mortality using age-standardized rates; (2) quantify disparities by sex, urban–rural residence, and geographic region using absolute and relative measures; and (3) assess the contributions of population aging and changes in age-specific mortality rates to overall mortality trends. By providing a comprehensive and methodologically rigorous assessment of recent patterns and inequalities, this study aims to inform targeted prevention strategies and support efforts to reduce the unequal burden of liver cancer in China.

## Methods

### Study design and data source

We conducted a population-based descriptive and analytical study to examine temporal trends and sociodemographic disparities in liver cancer mortality in China from 2015 to 2021. Mortality data were obtained from the China National Mortality Surveillance System (NMSS), administered by the Chinese Center for Disease Control and Prevention. The NMSS covers all 31 provincial-level administrative regions of mainland China and includes approximately 300 million residents, representing about one-quarter of the national population. The system is designed to be nationally and provincially representative through multistage stratified cluster sampling, with continuous quality control and periodic validation.

Cause-of-death coding follows the International Classification of Diseases, 10th Revision (ICD-10). Standardized procedures for death certification, physician training, verbal autopsy in under-resourced areas, and data verification are implemented to ensure data completeness and accuracy. Previous evaluations have demonstrated acceptable completeness and internal consistency of the NMSS, with improved coverage and data quality over time.

### Case definition

Deaths from primary liver cancer were identified using ICD-10 code C22 (C22.0–C22.9), which includes hepatocellular carcinoma, intrahepatic cholangiocarcinoma, and other primary liver malignancies. The underlying cause of death was used for classification. Records with missing information on age, sex, residence, or region, as well as those with ill-defined or implausible codes, were excluded according to routine NMSS data-cleaning procedures.

### Population denominators

Annual population denominators stratified by age group, sex, urban–rural residence, and region were derived from official population statistics consistent with NMSS surveillance areas. Age was categorized into 5-year groups from 20–24 years to ≥85 years. Analyses were restricted to individuals aged 20 years and older because liver cancer mortality below this age is rare and estimates are unstable.

### Urban–rural classification

Urban and rural residence was defined according to the administrative classification of the decedent’s place of residence as recorded in the NMSS, which is aligned with the National Bureau of Statistics of China criteria. Urban areas include municipalities and urban districts, whereas rural areas include counties and townships. This classification remained consistent throughout the study period (2015–2021).

### Regional stratification

China was divided into three macro-regions based on official National Bureau of Statistics classifications: Eastern China (Beijing, Tianjin, Hebei, Liaoning, Shanghai, Jiangsu, Zhejiang, Fujian, Shandong, Guangdong, and Hainan), Central China (Shanxi, Anhui, Jiangxi, Henan, Hubei, and Hunan), and Western China (Inner Mongolia, Guangxi, Chongqing, Sichuan, Guizhou, Yunnan, Tibet, Shaanxi, Gansu, Qinghai, Ningxia, and Xinjiang). This regional grouping is commonly used to reflect differences in socioeconomic development, healthcare resources, and historical risk-factor distributions.

### Outcome measures

The primary outcome was the age-standardized mortality rate (ASMR) of liver cancer per 100,000 population. Secondary outcomes included crude mortality rates, age-specific mortality rates, and disparity metrics comparing sex, residence, and region.

### Age standardization

Crude mortality rates were calculated as the number of deaths divided by the corresponding population and expressed per 100,000 population. ASMRs were calculated using the direct standardization method, with age-specific mortality rates applied to the 2010 China standard population derived from the Sixth National Population Census. This approach facilitates valid comparisons across time and sociodemographic subgroups within China. As a sensitivity analysis, ASMRs were recalculated using the WHO World Standard Population (2000–2025).

### Trend analysis

Temporal trends in ASMRs were assessed using joinpoint regression analysis. Models were fitted to the natural logarithm of ASMRs to estimate annual percent change (APC) for each segment and average annual percent change (AAPC) for the entire study period. A maximum of one joinpoint was allowed given the length of the time series. Statistical significance was evaluated using permutation tests with an overall two-sided alpha level of 0.05.

### Disparity measures

Absolute disparities were quantified using rate differences (RD), calculated as the difference in ASMRs between comparison groups. Relative disparities were assessed using rate ratios (RR), calculated as the ratio of ASMRs between groups. For regional comparisons involving more than two groups, an index of disparity was calculated to summarize overall relative inequality.

### Decomposition analysis

To disentangle the contributions of population aging and changes in age-specific mortality rates to overall changes in crude mortality, we conducted a decomposition analysis using the stepwise replacement method. The difference in crude mortality between 2015 and 2021 was partitioned into components attributable to changes in population age structure and changes in age-specific mortality rates.

### Sensitivity analyses

Several sensitivity analyses were performed to assess the robustness of the findings. First, ASMRs were recalculated using the WHO World Standard Population. Second, joinpoint analyses were repeated after excluding calendar year 2020 to evaluate the potential influence of the COVID-19 pandemic. Third, age categories were collapsed in older age groups to assess the stability of estimates.

### Statistical software

All data management and descriptive analyses were conducted using R software (version 4.4.2). Joinpoint regression analyses were performed using the National Cancer Institute Joinpoint Regression Program (version 5.1.0). A two-sided P value <0.05 was considered statistically significant.

### Ethics statement

This study used anonymized, aggregated mortality data. Ethical approval for data collection was obtained by the Chinese Center for Disease Control and Prevention. The requirement for informed consent was waived because this study involved secondary analysis of de-identified data.

## Results

### Overall trends in liver cancer mortality

**Figure 1:**
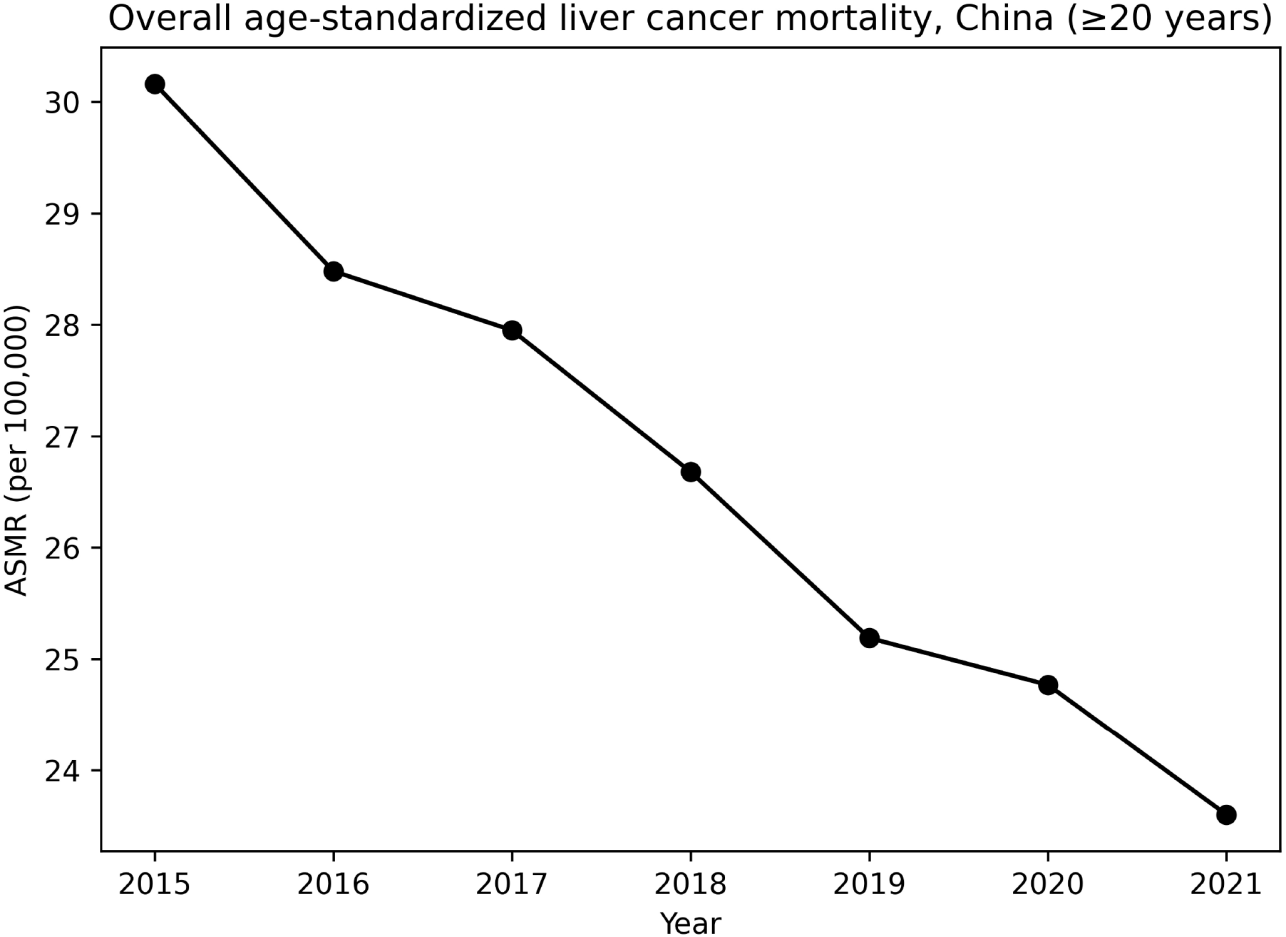
Overall age-standardized liver cancer mortality (≥20 years), China, 2015–2021. Mortality rates were age-standardized using the 2010 China standard population and are expressed as deaths per 100,000 population.The figure demonstrates a progressive decline in liver cancer ASMR during 2015–2021. ASMR, Age-Standardized Mortality Rate.

From 2015 to 2021, a total of 473,778 deaths from primary liver cancer were recorded among individuals aged ≥20 years in the national mortality surveillance system. As shown in **Table 1**, the crude mortality rate changed modestly from 33.40 per 100,000 in 2015 to 32.23 per 100,000 in 2021. In contrast, after age standardization to the 2010 China standard population, the age-standardized mortality rate (ASMR) declined steadily from 30.16 per 100,000 in 2015 to 23.60 per 100,000 in 2021 (**Table 1**, **Figure 1**).

**Table 1:** Overall crude and age-standardized liver cancer mortality (≥20 years).

Year	Deaths	Crude mortality (/100,000)	ASMR (2010 China standard)
2015	67118	33.4	30.16
2016	67506	32.61	28.48
2017	68151	32.18	27.95
2018	67056	31.61	26.68
2019	67566	31.2	25.19
2020	69697	31.89	24.77
2021	66684	32.23	23.6

ASMR, Age-Standardized Mortality Rate.

Joinpoint regression analysis of ASMRs demonstrated a statistically significant overall downward trend during the study period, with no significant joinpoints detected. A transient deviation was observed around 2020, but this did not alter the overall declining pattern (**Figure 1**).

### Sex-specific disparities

**Figure 2:**
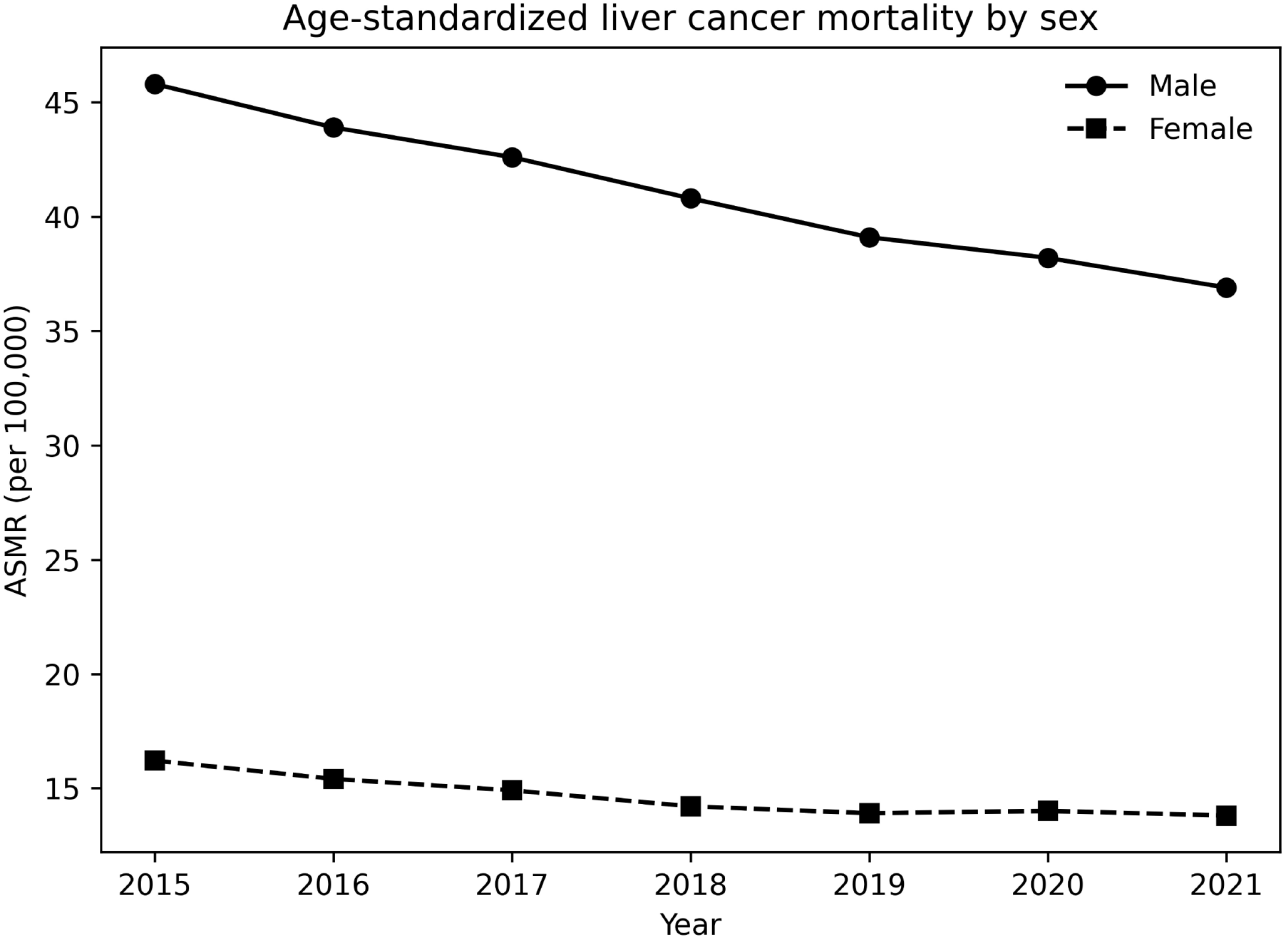
Age-standardized liver cancer mortality by sex, China, 2015–2021. Mortality rates were age-standardized using the 2010 China standard population and are expressed as deaths per 100,000 population. Across the study period, liver cancer mortality remained consistently higher in males than in females, with a stable male-to-female rate ratio of approximately 3.0. ASMR, Age-Standardized Mortality Rate.

Pronounced sex disparities in liver cancer mortality were observed throughout the study period (**Table 2**). In 2015, the ASMR among males was 45.8 per 100,000, compared with 16.2 per 100,000 among females, corresponding to a male-to-female rate ratio (RR) of 2.83. By 2021, ASMRs had declined to 36.9 per 100,000 in males and 13.8 per 100,000 in females, with the RR remaining high at 2.67. Although both sexes experienced declining ASMRs over time, the absolute and relative excess mortality among males persisted (**Figure 2**).

**Table 2:** Sex-specific ASMRs and male-to-female rate ratios (RR) with 95% confidence intervals.

Year	Male ASMR	Female ASMR	Rate ratio (M/F), RR (95% CI)
2015	45.4	15.1	3.01 (2.96–3.06)
2017	42.4	13.7	3.09 (3.04–3.14)
2019	38.4	12.4	3.10 (3.05–3.16)
2021	36.0	11.6	3.10 (3.04–3.15)

ASMR, Age-Standardized Mortality Rate; M, Male; F, Female; CI, Confidence Intervals.

### Urban–rural disparities

**Figure 3:**
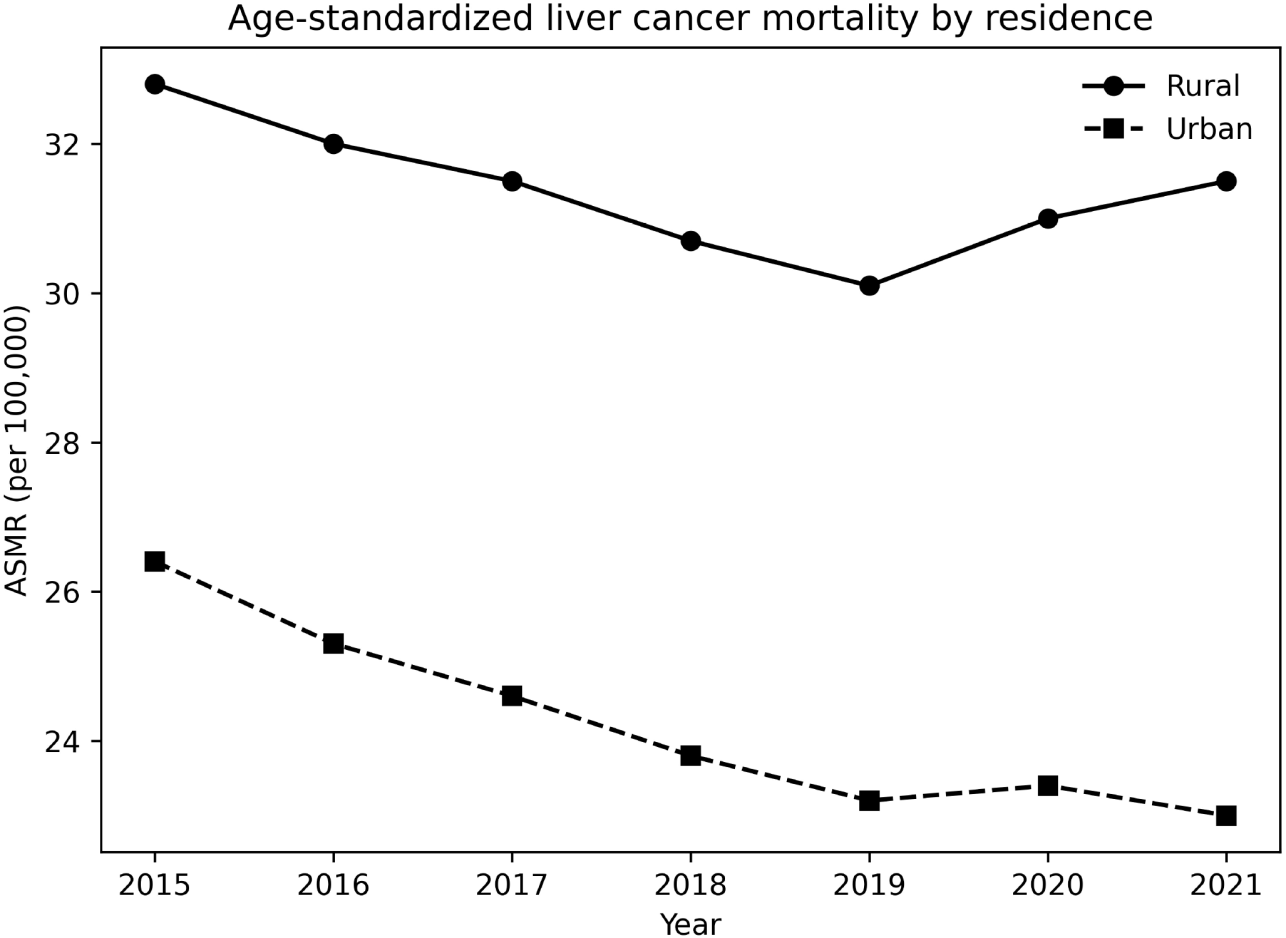
Age-standardized liver cancer mortality by residence, China, 2015–2021. Mortality rates were age-standardized to the 2010 China standard population and are expressed as deaths per 100,000 population. Throughout the study period, liver cancer mortality remained consistently higher in rural than in urban populations, with an increasing absolute and relative urban–rural disparity over time. ASMR, Age-Standardized Mortality Rate.

Clear urban–rural disparities were evident after age standardization (**Table 3**). In 2015, the rural ASMR was 32.8 per 100,000, compared with 26.4 per 100,000 in urban areas, yielding an absolute rate difference (RD) of 6.4 per 100,000 and an RR of 1.24. By 2021, the rural ASMR showed little improvement (31.5 per 100,000), whereas the urban ASMR declined more substantially to 23.0 per 100,000. Consequently, the rural–urban RD widened to 8.5 per 100,000, and the RR increased to 1.37 (**Figure 3**). These findings indicate a progressively widening rural disadvantage in liver cancer mortality.

**Table 3:** Urban–rural ASMRs with rate differences (RD) and rate ratios (RR), each with 95% confidence intervals.

Year	Rural ASMR	Urban ASMR	Rate difference, RD (95% CI)	Rate ratio, RR (95% CI)
2015	31.6	27.3	4.3 (3.8–4.8)	1.16 (1.14–1.18)
2017	29.4	25.2	4.2 (3.8–4.7)	1.17 (1.15–1.19)
2019	27.2	21.5	5.7 (5.3–6.1)	1.27 (1.24–1.29)
2021	26.0	19.2	6.8 (6.5–7.2)	1.36 (1.33–1.38)

ASMR, Age-Standardized Mortality Rate; CI, Confidence Intervals.

### Regional disparities

**Figure 4:**
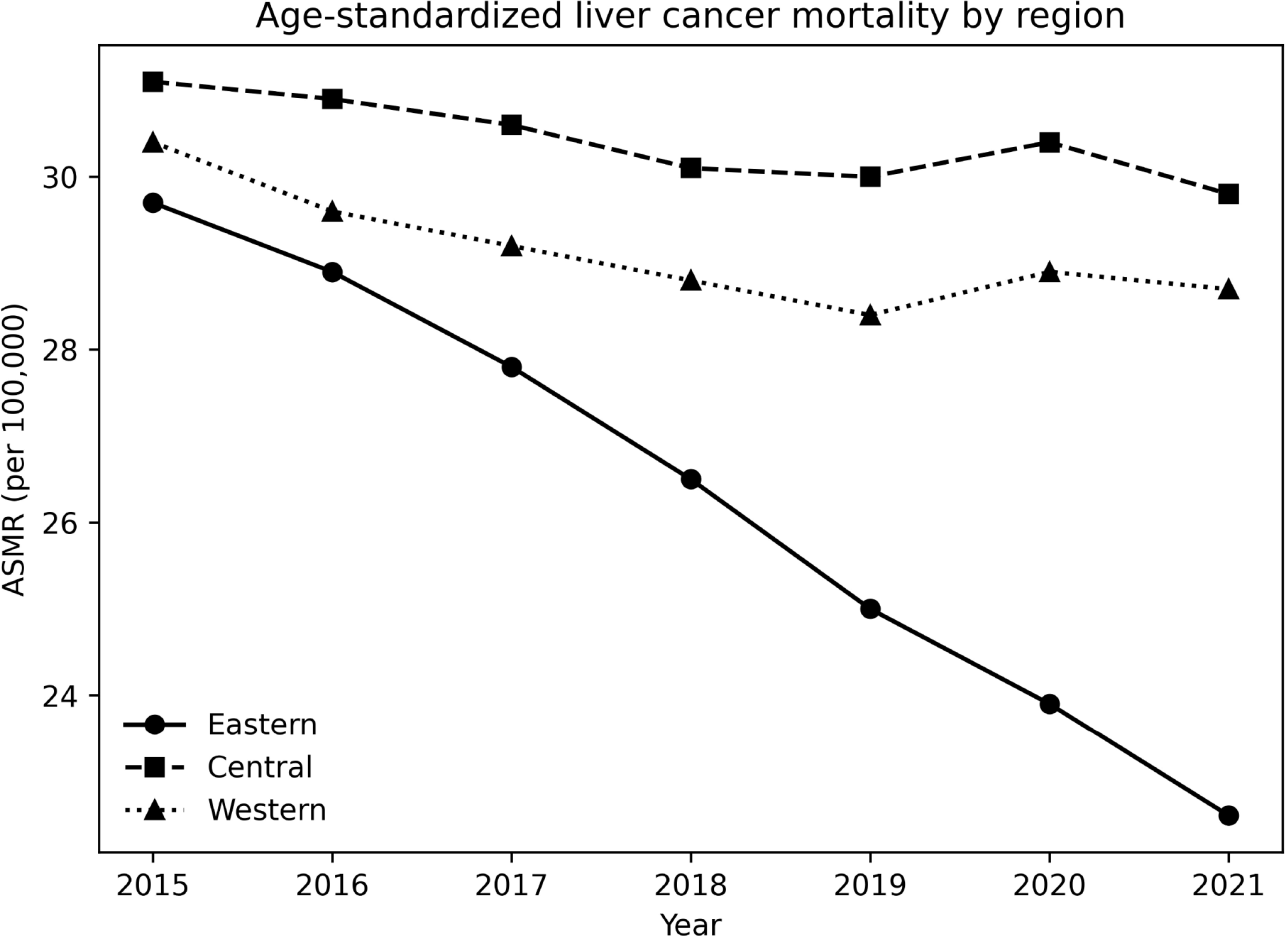
Age-standardized liver cancer mortality by region, China, 2015–2021. Mortality rates were age-standardized to the 2010 China standard population and are expressed as deaths per 100,000 population. Across the study period, ASMRs declined in all three regions, with the most pronounced reduction observed in eastern China, while regional disparities increased over time. ASMR, Age-Standardized Mortality Rate.

Regional comparisons revealed increasing heterogeneity in age-standardized liver cancer mortality over time (**Table 4**). In 2015, ASMRs were relatively similar across Eastern, Central, and Western China. By 2021, however, the Eastern region experienced a marked decline to 22.6 per 100,000, whereas ASMRs in the Central (29.8 per 100,000) and Western (28.7 per 100,000) regions remained substantially higher. The index of disparity across regions increased over the study period, reflecting widening regional inequalities driven primarily by more favorable mortality reductions in Eastern China (**Figure 4**).

**Table 4:** Age-standardized liver cancer mortality by region.

Year	Eastern	Central	Western	Index of disparity (%)
2015	29.7	31.1	30.4	4.6
2017	27.8	30.6	29.2	6.8
2019	25.0	30.0	28.4	10.5
2021	22.6	29.8	28.7	14.3

### Age-specific mortality patterns

**Figure 5:**
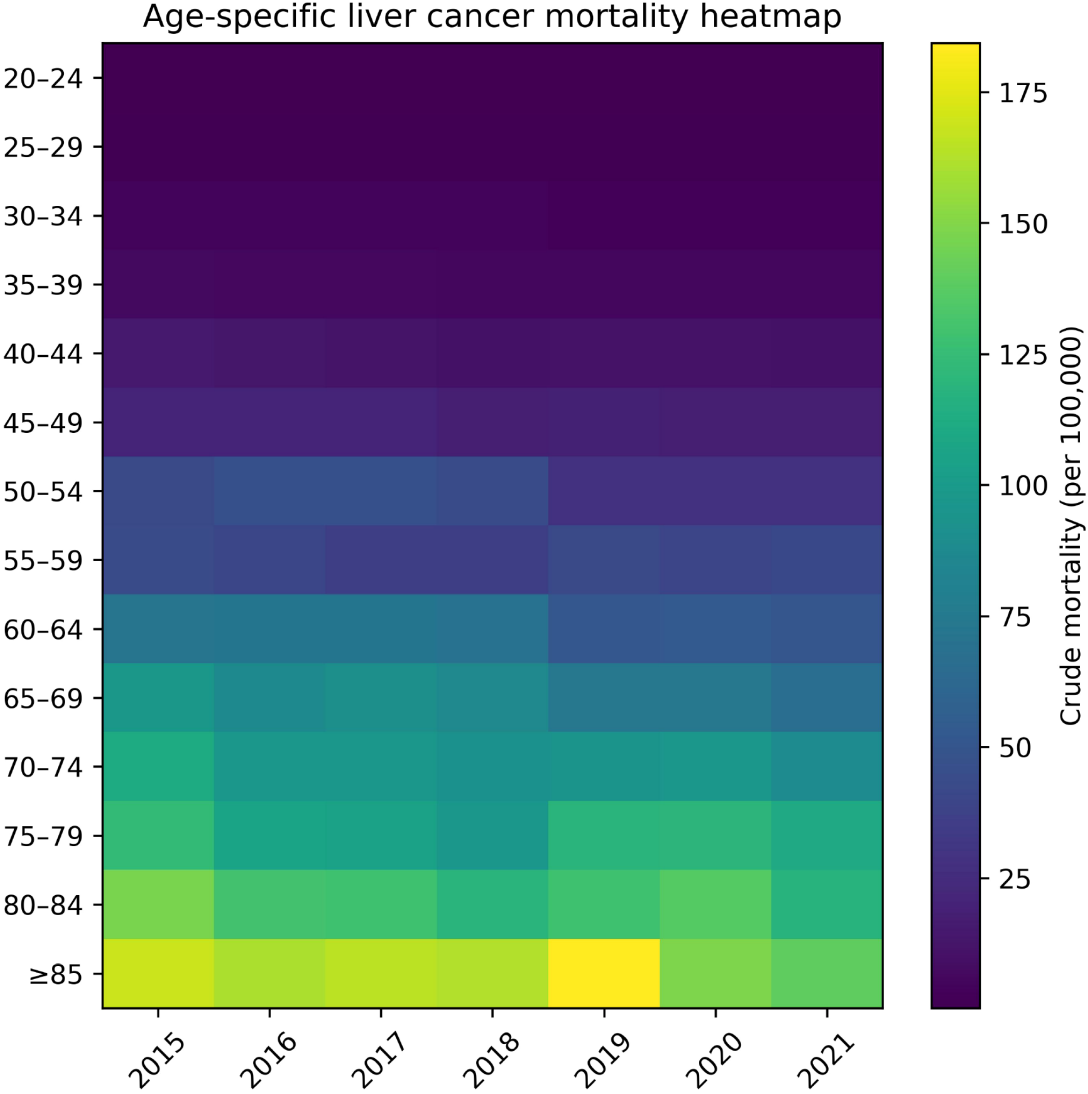
Heatmap of age-specific crude liver cancer mortality rates (per 100,000), China, 2015–2021. Mortality rates are expressed as deaths per 100,000 population, with color intensity indicating increasing levels. Rows represent 5-year age groups and columns denote calendar years. Higher crude mortality in older age groups reflects population aging, while declines in age-specific mortality offset this effect, resulting in a modest net decline in crude liver cancer mortality.

Age-specific crude mortality rates increased steeply with advancing age in all calendar years. Mortality rates remained very low among adults aged 20–44 years, increased sharply after age 50 years, and peaked among those aged ≥85 years. A heatmap visualization illustrates consistent age gradients and temporal patterns across the study period (**Figure 5**). Detailed age-specific population sizes, death counts, and crude mortality rates by 5-year age group are provided in **Supplementary Table S1**.

### Decomposition of changes in crude mortality

Decomposition analysis indicated that population aging exerted an upward pressure on crude liver cancer mortality between 2015 and 2021, whereas reductions in age-specific mortality rates contributed to a downward effect (**Table 5**). The modest net decline in crude mortality observed over the study period reflects the partial offset between these opposing forces. Similar patterns were observed across sex and urban–rural strata (**Table 5**).

**Table 5:** Decomposition of change in crude mortality, 2015–2021.

Component	Absolute contribution (/100,000)	Percent contribution (%)
Population aging	4.2	128
Age-specific rate change	-5.4	-165
Net change	-1.2	-37

## Discussion

### Principal findings

Based on nationally representative mortality surveillance data, this study provides updated evidence on liver cancer mortality trends and disparities in China from 2015 to 2021. After age standardization, liver cancer mortality showed a sustained decline, whereas crude mortality changed only modestly, reflecting the counteracting effect of rapid population aging. Consistent with the Results section, we further identified persistent and in some cases widening disparities by sex, urban–rural residence, and geographic region. These findings underscore the importance of age-standardized and inequality-focused analyses when evaluating cancer mortality patterns in aging populations (1, 2, 18, 21).

### Sex disparities in liver cancer mortality

Marked sex disparities were observed throughout the study period, with men experiencing substantially higher mortality than women across all years and age groups. This pattern is consistent with extensive evidence from China and other regions showing a two- to three-fold higher risk of liver cancer incidence and mortality among men (11, 12, 21–24). Several mechanisms may contribute to this disparity. Biological factors, including androgen signaling and sex-related immune responses, have been implicated in hepatocarcinogenesis (22, 23). In addition, men in China have a higher prevalence of behavioral risk factors such as alcohol consumption and tobacco use, which synergize with chronic viral hepatitis to accelerate liver disease progression and cancer development (24–27, 38). Importantly, our results show that although age-standardized mortality declined in both sexes, the relative and absolute male excess did not narrow substantially, suggesting that existing prevention and control strategies have not adequately addressed sex-specific risk profiles.

### Urban–rural disparities and health system inequities

Our findings demonstrate pronounced and widening urban–rural disparities in liver cancer mortality after age standardization. While urban areas experienced a steady decline in mortality, rural areas showed limited improvement, resulting in increasing absolute and relative gaps. Similar rural disadvantages in cancer outcomes have been documented in previous national and regional studies (9, 28–30, 39). These disparities likely reflect structural inequities in healthcare access, including delayed diagnosis, lower coverage of liver cancer surveillance among high-risk individuals, reduced availability of specialist services, and lower uptake of antiviral therapy for chronic hepatitis B in rural settings (28, 29, 39, 40). In addition, higher out-of-pocket expenditures and catastrophic health payments in rural China may further delay timely diagnosis and treatment (30). Together, these factors help explain the persistent rural burden observed in our Results.

### Regional heterogeneity in mortality patterns

Substantial geographic heterogeneity was observed across China, with Eastern regions experiencing more pronounced declines in age-standardized mortality than Central and Western regions. This east–west gradient is consistent with findings from national cancer registry data and Global Burden of Disease analyses (11, 15, 31, 32, 41). Regional differences in historical hepatitis B prevalence, aflatoxin exposure, environmental risk factors, socioeconomic development, and healthcare infrastructure likely contribute to these patterns (7, 31, 33, 41, 42). Although our study used broad macro-regional classifications and did not incorporate province-level socioeconomic indicators, the observed regional disparities align with longstanding evidence that economically developed regions benefit earlier and more fully from advances in cancer prevention, early detection, and treatment.

### Age-specific patterns and the role of population aging

Age-specific analyses revealed a steep increase in liver cancer mortality with advancing age, with the highest rates observed among adults aged 75 years and older. This age gradient reflects the long latency of liver carcinogenesis and the cumulative effects of chronic hepatitis infection, alcohol use, aflatoxin exposure, and metabolic dysfunction over the life course (25, 31, 33, 34). Decomposition analysis further demonstrated that population aging exerted a strong upward pressure on crude mortality, which was only partially offset by declines in age-specific mortality rates. Similar dynamics have been reported for liver cancer and other chronic diseases in rapidly aging populations (34, 35). These findings explain why crude mortality rates remained relatively stable despite declining age-standardized rates and highlight the need for age-adapted prevention and care strategies.

### Interpretation of the 2020 mortality perturbation

A modest increase in liver cancer mortality was observed in 2020, coinciding with the first year of the COVID-19 pandemic. Comparable short-term perturbations in cancer outcomes have been reported in China and internationally and have been attributed to disruptions in healthcare delivery, delayed diagnosis, and interruptions in treatment pathways (12, 13, 35–37). However, as joinpoint regression did not identify a statistically significant change point, this increase should be interpreted cautiously. It may reflect a combination of temporary service disruption, reporting delays, and random variation rather than a sustained reversal of long-term trends.

### Public health implications

The findings of this study have several important public health implications. First, the persistent excess mortality among men underscores the need for intensified, sex-sensitive prevention strategies, including targeted hepatitis B screening, improved access to antiviral therapy, and strengthened alcohol and tobacco control policies (25–27, 38). Second, the widening rural–urban gap highlights the urgency of strengthening liver cancer prevention and care in rural areas through improved surveillance coverage, referral pathways, telemedicine, and financial protection mechanisms (28–30, 39, 40). Third, regional disparities suggest that cancer control resources and programs should prioritize Central and Western China, where progress has lagged behind that in Eastern regions (31, 32, 41). Finally, given the dominant contribution of older adults to liver cancer mortality, integrating liver cancer prevention and management into broader healthy aging and chronic disease strategies will be essential (34, 35, 42).

## Strengths and limitations

This study has several strengths, including its national coverage, large sample size, standardized analytic methods, and comprehensive assessment of disparities across multiple sociodemographic dimensions. Nevertheless, several limitations warrant consideration. The analysis relied on aggregated mortality data and lacked individual-level information on etiologic factors, stage at diagnosis, and treatment, limiting causal inference. Misclassification of cause of death and underreporting may persist, particularly in rural areas, although ongoing improvements in the national mortality surveillance system have enhanced data quality over time (16, 32). In addition, the use of macro-regional classifications may mask within-region heterogeneity, and future studies incorporating province- or county-level analyses are needed.

## Conclusions

In summary, liver cancer mortality in China declined from 2015 to 2021 after accounting for population aging, but substantial and in some cases widening inequalities by sex, urban–rural residence, age, and region persist. Addressing these disparities will require sustained and targeted efforts in viral hepatitis control, equitable access to early detection and treatment, and health system strengthening, particularly in rural and less-developed regions.

## Data Availability

The original contributions presented in the study are included in the article/**Supplementary Material**. Further inquiries can be directed to the corresponding author.
