# Supplementary material for: Socio-demographic disparities in liver cancer mortality in China: a national analysis from 2015 to 2021
Source: Front Oncol. 2026 Feb 3;16:1757881. doi: 10.3389/fonc.2026.1757881 (PMC12909251; doi:10.3389/fonc.2026.1757881)
Supplement: Supplementary file 1 [file DataSheet1.pdf]

**Supplementary Table S1. Age-specific liver cancer mortality by age group (≥20 years), China, 2015–2021**

Crude age-specific mortality rates were calculated as deaths divided by population and expressed per 100,000 population. Age groups are presented in 5-year intervals. These detailed age-specific data support the calculation of age-standardized mortality rates (ASMRs) using the 2010 China standard population.

| Year | Age group (years) | Population | Deaths | Crude mortality (per 100,000) |
|---|---|---|---|---|
| 2015 | 20–24 | 22895079 | 121 | 0.53 |
| 2015 | 25–29 | 18670345 | 301 | 1.61 |
| 2015 | 30–34 | 15611678 | 671 | 4.3 |
| 2015 | 35–39 | 19793806 | 1405 | 7.1 |
| 2015 | 40–44 | 22967145 | 3386 | 14.74 |
| 2015 | 45–49 | 26394480 | 5582 | 21.15 |
| 2015 | 50–54 | 16034245 | 6787 | 42.33 |
| 2015 | 55–59 | 18285929 | 7964 | 43.55 |
| 2015 | 60–64 | 14053811 | 9985 | 71.05 |

| | | | | |
|---|---|---|---|---|
| 2015 | 65–69 | 9032555 | 8808 | 97.51 |
| 2015 | 70–74 | 6728141 | 7551 | 112.23 |
| 2015 | 75–79 | 5350578 | 6600 | 123.35 |
| 2015 | 80–84 | 3320829 | 4893 | 147.34 |
| 2015 | ≥85 | 1817600 | 3064 | 168.57 |
| 2021 | 20–24 | 13887555 | 43 | 0.31 |
| 2021 | 25–29 | 15366280 | 157 | 1.02 |
| 2021 | 30–34 | 21585599 | 591 | 2.74 |
| 2021 | 35–39 | 17540645 | 1027 | 5.85 |
| 2021 | 40–44 | 18030728 | 1950 | 10.81 |
| 2021 | 45–49 | 22055010 | 3974 | 18.02 |
| 2021 | 50–54 | 23555219 | 6514 | 27.65 |
| 2021 | 55–59 | 19932475 | 8239 | 41.33 |
| 2021 | 60–64 | 15022915 | 7589 | 50.52 |
| 2021 | 65–69 | 15439854 | 10475 | 67.84 |
| 2021 | 70–74 | 10328564 | 9209 | 89.16 |
| 2021 | 75–79 | 6553548 | 7275 | 111.01 |

| | | | | |
|---|---|---|---|---|
| 2021 | 80–84 | 4550606 | 5378 | 118.18 |
| 2021 | ≥85 | 3058720 | 4263 | 139.37 |
